# Supplementary material for: Integrated UAV photogrammetry and SMRM constitutive modeling for spatial stability assessment of layered rock slopes
Source: PLoS One. 2026 Apr 30;21(4):e0341174. doi: 10.1371/journal.pone.0341174 (PMC13132238; doi:10.1371/journal.pone.0341174)
Supplement: Attachment — Submitted filename: Response to Reviewer.docx [file pone.0341174.s003.docx]

**Response to Editor**

**Dear Editor**

Thank you very much for your email concerning our manuscript entitled " *Integrated UAV Photogrammetry and SMRM Constitutive Modeling for Spatial Stability Assessment of Layered Rock Slopes*” (ID: PONE-D-25-24746). We have carefully considered the points raised by the reviewers, and have made the following responses and changes. We deem *PLOS ONE a*s a very excellent journal and hope the submitted revision can be accepted by this international journal.

Here, we would like to thank the reviewers for their insightful comments, and very much appreciate your patience, time and efforts in handling the reviewing process.

Best regards,

All authors

**Issues 1:**“ In your Methods section, please provide additional information regarding the permits you obtained for the work. Please ensure you have included the full name of the authority that approved the field site access and, if no permits were required, a brief statement explaining why. ”

**Authors' responses:**

Thank you for your careful review and valuable comments on our revised manuscript. We have carefully addressed each of your concerns and made corresponding revisions. Below is our response to your specific comment:

The following sentence has been added in Chapter 2.1 :

"No specific permits were required for this study because it did not involve human participants, animal subjects, endangered or protected species, or access to protected areas. All data collected and analyzed were from publicly available sources."

This study did not require specific permits for the described research activities, in accordance with key authorities:

The *PLOS ONE Author Guidelines on Ethics, Consent, and Permissions* (retrieved from: https://journals.plos.org/plosone/s/submission-guidelines#loc-ethics-consent-and-permissions):

- Humans (live or tissue), including studies that are observational, survey-based, or include any personal data
- Animals (live or tissue), including observational studies
- Cell lines that are not commercially available
- Field sampling
- Potential biosafety implications

Thus, no permits were required to conduct this study.

**Issues 2:**“ We note that the grant information you provided in the ‘Funding Information’ and ‘Financial Disclosure’ sections do not match. ”

**Authors' responses:**

Founder: Department of transportation of Zhejiang Province;

Award Number: 2024-GCKY-01

Grant Recipient: Huagang Shan

**Issues 3:**“ Thank you for updating your data availability statement. You note that your data are available within the Supporting Information files, but no such files have been included with your submission. At this time we ask that you please upload your minimal data set as a Supporting Information file, or to a public repository such as Figshare or Dryad.
Please also ensure that when you upload your file you include separate captions for your supplementary files at the end of your manuscript.
As soon as you confirm the location of the data underlying your findings, we will be able to proceed with the review of your submission.”

**Authors' responses:**

Thank you for your follow-up regarding the data availability.We have stored the data in the Figshare public repository.

We have now revised the Data Availability Statement in our manuscript to accurately reflect the location of the data. The statement now reads:

“All relevant data are within the paper and its Supporting Information files.”

Additionally, as requested, we have added a "Supporting Information" section at the end of the manuscript file that includes captions for these datasets:

Supporting information

S1 File.Information about rock material parameters for slope stability analysis.

10.6084/m9.figshare.29377223

(XLSX)

S2 File.Information about Statistical window structure plane parameter table.

DOI:10.6084/m9.figshare.29377235

(XLSX)

The minimal data set required to replicate all study findings is fully accessible via the provided DOIs.

We hope these revisions fully address your concerns. Thank you for your attention to this matter.

**Issues 4:**“Can you please upload an additional copy of your revised manuscript that does not contain any tracked changes or highlighting as your main article file.  This will be used in the production process if your manuscript is accepted.  Please amend the file type for the file showing your changes to Revised Manuscript w/tracked changes. Please follow this link for more information: [http://blogs.PLOS.org/everyone/2011/05/10/how-to-submit-your-revised-manuscript/](http://blogs.plos.org/everyone/2011/05/10/how-to-submit-your-revised-manuscript/) ”

**Authors' responses:**

Thank you for your email regarding the submission of our revised manuscript.

We have now prepared and uploaded the two required files as instructed:

(1) A marked-up copy of your manuscript that highlights changes made to the original version. You should upload this as a separate file labeled 'Revised Manuscript with Track Changes'.

(2) An unmarked version of your revised paper without tracked changes. You should upload this as a separate file labeled 'Manuscript'.

We believe we have fully addressed all points raised by the reviewers, and we have detailed our responses in the point-by-point response letter.

Thank you for handling our manuscript.

**Issues 5:**“We note that Figures 1, 2, 3, 4, 5 and 7 in your submission contain [map/satellite] images which may be copyrighted. All PLOS content is published under the Creative Commons Attribution License (CC BY 4.0), which means that the manuscript, images, and Supporting Information files will be freely available online, and any third party is permitted to access, download, copy, distribute, and use these materials in any way, even commercially, with proper attribution. For these reasons, we cannot publish previously copyrighted maps or satellite images created using proprietary data, such as Google software (Google Maps, Street View, and Earth). For more information, see our copyright guidelines: http://journals.plos.org/plosone/s/licenses-and-copyright. ”

**Authors' responses:**

Thank you for your detailed guidance on ensuring copyright compliance for the figures in our manuscript. We deeply appreciate your patience and have carefully addressed the concerns raised regarding Figures 1, 2, 3, 4, 5, and 7, ensuring full alignment with PLOS ONE’s CC BY 4.0 license requirements. Below is our detailed response for each figure:

1. Figure 1 (Map/Satellite Image)

In the previous revision, we replaced the original figure with a map generated from Natural Earth (http://www.naturalearthdata.com/), a public domain resource explicitly released under no copyright restrictions. To further clarify compliance:

“We have added explicit source information in the figure caption: Fig. 1 Geographic location of the study area. Map generated using Natural Earth data, No copyright restrictions apply.”

2. Figures 2, 3, and 7 (Field Photographs)

These figures are original photographs taken by the authors during field investigations at the Kezhu Expressway. To eliminate ambiguity:

We have updated their captions to explicitly state: “Fig.2 Images of the study area: (a) Aerial view of the study area (b) Photo of layered rock survey (c) Engineering geological cross-section. Original photograph taken by the authors during fieldwork at the Kezhu Expressway. Copyright held by the authors, released under the Creative Commons Attribution License (CC BY 4.0).” (Same format applied to Figures 3 and 7.)

As original creators, we confirm full rights to license these images under CC BY 4.0, permitting unrestricted use with proper attribution.

3. Figure 4, 5

Thank you for your comment. We confirm that these figures are original and created solely by the authors. They do not contain screenshots or any copyrighted elements of software interfaces.

The figures depict the scientific results and workflow outputs generated through our use of various tools (e.g., processed point clouds, 3D models, and simulation results). The copyright of these scientific findings belongs to us as the creators, and we release them under the CC BY 4.0 license.

|  |  |
| --- | --- |
| **Fig. 1 Geographic location of the study area**  **Map generated using Natural Earth data, No copyright restrictions apply.** | |

**Fig.2 Images of the study area: (a) Aerial view of the study area (b) Photo of layered rock survey (c) Engineering geological cross-section**

**(Original photograph taken by the authors during fieldwork at the Kezhu Expressway. Copyright held by the authors, released under the Creative Commons Attribution License (CC BY 4.0).)**

**Fig.3 Drone flight preparation and on-site work**

**(Original photograph taken by the authors during fieldwork at the Kezhu Expressway. Copyright held by the authors, released under the Creative Commons Attribution License (CC BY 4.0).)**

|  |  | |  |
| --- | --- | --- | --- |
| **(a) Three-dimensional image model** | **(b) Point cloud decimation** | | **(c) Point cloud meshing** |
|  |  | |  |
| **(d) Mesh** | **(e) Surface** | | **(f) Surface solid** |
|  | |  | |
| **（g） Griddle mesh subdivision** | | **（h） Flac numerical model** | |
| **Fig.4 “CC-Griddle-Flac3d” modeling**  Original model created by the authors. Copyright held by the authors, released under the Creative Commons Attribution License (CC BY 4.0). | | | |

**Fig.5 3D numerical model of the slope**

**Original model generated by the authors. Copyright held by the authors, released under the Creative Commons Attribution License (CC BY 4.0).**

**Fig.7 The area to be measured in the field**

**(Original photograph taken by the authors during fieldwork at the Kezhu Expressway. Copyright held by the authors, released under the Creative Commons Attribution License (CC BY 4.0).)**
