# Supplementary material for: Integrated UAV photogrammetry and SMRM constitutive modeling for spatial stability assessment of layered rock slopes
Source: PLoS One. 2026 Apr 30;21(4):e0341174. doi: 10.1371/journal.pone.0341174 (PMC13132238; doi:10.1371/journal.pone.0341174)
Supplement: Attachment — Submitted filename: Response to Reviewer_new.docx [file pone.0341174.s004.docx]

**Response to Editor**

**Dear Editor**

Thank you very much for your email concerning our manuscript entitled " *Integrated UAV Photogrammetry and SMRM Constitutive Modeling for Spatial Stability Assessment of Layered Rock Slopes*” (ID: PONE-D-25-24746). We have carefully considered the points raised by the reviewers, and have made the following responses and changes. We deem *PLOS ONE a*s a very excellent journal and hope the submitted revision can be accepted by this international journal.

Here, we would like to thank the reviewers for their insightful comments, and very much appreciate your patience, time and efforts in handling the reviewing process.

Best regards,

All authors

**Issues 1:**“Fig.1b/Fig.2C: The image resolution is insufficient.”

**Authors' responses:**

We appreciate the reviewer's attention to detail and agree that improving the image resolution is necessary for clarity. Enclosed are the high-resolution versions of Figures 1b and 2C.

**Fig. 1 Geographic location of the study area**

**Fig.2 Images of the study area: (c) Engineering geological cross-section**

**Issues 2:**“Numerical calculations are based on the actual slope topography. Since the area has dense vegetation, please specify whether vegetation removal was performed during point cloud processing.”

**Authors' responses:**

Thank you for your valuable comment. In response to your inquiry, we have clarified in the revised manuscript (lines 152-155) that vegetation removal was performed during point cloud processing.

“The Cloth Simulation Filter (CSF) method was used to remove vegetation data and retain ground information. This process separated vegetation points from ground points, ensuring that the numerical calculations considered only terrain features.”

**Issues 3:**“The modeling process description in Section 3 is overly detailed. It is recommended to use a flowchart for illustration.”

**Authors' responses:**

Thank you for your valuable feedback. We agree that the modeling process description in Section 3 was too detailed, and we have revised the section to provide a more concise overview. We have simplified the text and added a flowchart(Fig. 3) to illustrate the key steps in the modeling process more clearly. The updated Section 3 now focuses on the essential steps and provides an easier-to-follow representation of the methodology.

**Issues 4:**“The meaning of Em1111 in Formula 3 is unclear.”

**Authors' responses:**

Thank you for pointing this out. We have revised Formula 3 by changing Em1111 to Em, and we have added a clarification to the text: "where Em is the elastic modulus of the rock mass."

**Issues 5:**“The grouping in Figures 4 and 5 lacks annotations.”

**Authors' responses:**

Figure 4 already contains subplot labels; Figure 5 now has additional annotations for each rock layer.

**Fig.6 3D numerical model of the slope**

**Issues 6:**“In Table 1, the unit for tensile strength is incorrect.”

**Authors' responses:**

The unit in column 4 of Table 1 has been changed from “Pa” to “MPa”.

**Issues 7:**“Line 372: The expression "The bedding orientation is 332°∠24°" is unclear.”

**Authors' responses:**

We agree that the single-angle notation may be unfamiliar to some readers.

The sentence (now line 321) has been rewritten as:

“The bedding plane orientation is 332° (dip Angle) ∠ 24° (dip). ”

**Issues 8:**“For Table 2, please clarify whether the structural surfaces are simulated using standard contact models. If so, provide specific parameters and value determination methods.”

**Authors' responses:**

No, a user-defined constitutive model (SMRM model) was developed to simulate the mechanical behavior of structural surfaces in this study. The detailed formulation and validation of the SMRM model are presented in Section 4.

The geometric parameters of the structural surfaces (e.g., dip angle, dip direction, trace length, and normal density) were obtained from field measurements using the sampling window method.

The strength parameters (cohesion C and friction angle φ) were empirically determined based on engineering experience and typical values for moderately weathered tuffaceous sandstone, as shown in Table 2.

**Issues 9:**“Regarding the displacement nephograms in Figures 8 and 9, please specify: a) What processing they are based on. b) The cause of displacement generation.”

**Authors' responses:**

a) The following sentence has been added in Section 5.3.1 (lines 353–355):

The total displacement magnitudes in Figures 8 and 9 were extracted from the centroids of all tetrahedral elements in FLAC3D and then mapped into nephograms using volumetric averaging.

b) The subsequent sentence (lines355–357) now reads:

“Displacement in both models develops after gravity loading; specifically, the deformation shown in Fig. 10 (SMRM model) is dominated by shear slip along the gently dipping structural planes (24°).”
